# Factors Predicting Glycemic Control in Middle-Aged and Older Adults With Type 2 Diabetes

**Published:** 2009-12-15

**Authors:** Ching-Ju Chiu, Linda A. Wray

**Affiliations:** Department of Biobehavioral Health; The Pennsylvania State University, University Park, Pennsylvania

## Abstract

**Introduction:**

Few studies have prospectively assessed the explanatory effects of demographics, clinical conditions, treatment modality, and general lifestyle behaviors on glycemic control in large heterogeneous samples of middle-aged and older adults with type 2 diabetes. We hierarchically examined these factors, focused especially on the effects of modifiable factors (ie, general lifestyle behaviors), and compared predictive patterns between middle-aged and older adults.

**Methods:**

We used nationally representative data from the 1998 and 2000 Health and Retirement Study (HRS) and the HRS 2003 Diabetes Study. We analyzed data from 379 middle-aged adults (aged 51-64 y) and 430 older adults (aged ≥65 y) who self-reported having type 2 diabetes at baseline.

**Results:**

Among middle-aged adults, demographic factors and clinical conditions were the strongest predictors of hemoglobin A1c (HbA1c) levels. However, among older adults, treatment modality (diet only, oral medication, or insulin only or in combination with other regimens) significantly affected HbA1c levels. Lifestyle (physical activity, smoking, drinking, and body weight control), independent of the effects of demographics, clinical conditions, and treatment modality, significantly affected HbA1c levels. An increase of 1 healthy behavior was associated with a decrease in HbA1c levels of more than 1 percentage point.

**Conclusion:**

Our findings provide support for current diabetes guidelines that recommend a lifestyle regimen across the entire span of diabetes care and highlight the need to help both sociodemographically and clinically disadvantaged middle-aged adults with type 2 diabetes as well as older adults who exhibit poor adherence to medication recommendations to achieve better glycemic control.

## Introduction

Type 2 diabetes affects a large number of middle-aged and older adults in the United States. More than 16 million adults in the United States and 200 million people worldwide have the disease (1). One of the primary goals of diabetes management is to lower blood glucose levels because it is well established that improved glycemic control delays the onset and retards the progression of microvascular and macrovascular complications (2). However, many US adults (40%-60%) with type 2 diabetes have poor glycemic control (3,4). Understanding the factors that contribute to glycemic control is key to developing more effective treatment and identifying the needs of adults with type 2 diabetes.

Lifestyle behaviors are postulated to play a role in glycemic control, and their effects on glycemic control have been given increasing attention in the past decade. However, most studies in this area have focused only on the effects of diabetes-specific self-management behaviors on glycemic control (5-7). Furthermore, other studies that have demonstrated the effects of general health behaviors on hemoglobin A1c (HbA1c) levels have focused on a single lifestyle behavior, such as exercise or weight control (8,9). Although findings from these studies have been encouraging, most studies have examined changes in HbA1c levels among homogeneous samples within a short period after the strictly controlled intervention. The effectiveness of general lifestyle behaviors on long-term glycemic control among adults from heterogeneous backgrounds, with differing clinical conditions, and who use different forms of treatment remains uncertain.

The influences of demographics, clinical conditions, and treatment adherence on glycemic control have also been documented. Previous studies have suggested that minority groups (eg, African Americans, Hispanics, American Indians, Pacific Islanders) (10,11) and adults who have had diabetes for a long time, who have comorbidities (12,13), or who use insulin or multiple oral agents have high HbA1c levels (12,14). However, longitudinal evidence regarding the explanatory effects of these variables on glycemic control from large heterogeneous national samples is sparse. Because these factors have not been examined comprehensively, whether clinical conditions explain the racial/ethnic disparities or whether the effect of clinical conditions on glycemic control differs among people from different demographic backgrounds is unclear.

Furthermore, there has been little investigation of the predictive factors of glycemic control in midlife and older age. As suggested by cumulative advantage/disadvantage theory, age has a modifying effect on many explanatory factors in health outcomes (15). Therefore, we hypothesized that distinct predictive patterns of glycemic control exist between middle-aged and older adults. The effects of dietary therapy on glycemic control in clinical settings generally deteriorate over time (14). However, current literature does not answer the question of whether older adults are less sensitive to other modifiable factors, such as health behaviors.

We prospectively examined the effects of general lifestyle behaviors (physical activity, smoking, drinking, and weight control) on HbA1c levels, controlling for documented correlates, including demographic characteristics (4,16,17), clinical conditions (12,13), and treatment modalities (14,18). We studied a nationally representative sample of middle-aged and older adults with type 2 diabetes. We demonstrate the complex explanatory effects of each factor on glycemic control by using a heterogeneous sample, longitudinal design, and hierarchical regression analysis. We tested the hierarchical models among middle-aged adults and older adults separately to examine whether distinct predictive patterns between middle-aged and older adults exist.

## Methods

### Participants

Our study sample was 379 middle-aged adults (aged 51-64 y) and 430 older adults (aged ≥65 y) who 1) in the 1998 Health and Retirement Study (HRS) interview self-reported having diagnosed type 2 diabetes after age 34 and 2) returned valid blood spot tests for HbA1c in the 2003 HRS Diabetes Study.

The HRS is an ongoing biennial survey that was initiated in 1992 to track the health status and retirement plans of community-dwelling middle-aged and older US adults and that oversamples Hispanic Americans and African Americans. Details about recruitment procedures and characteristics of the participants in the HRS interview are described elsewhere (19). The HRS 2003 Diabetes Study followed adults who self-reported diagnosed diabetes in the 2002 or earlier HRS interviews and collected data on various diabetes-related psychological, behavioral, and clinical measures. We weighted data to make the study sample representative of US middle-aged and older adults with type 2 diabetes who were born in 1947 or earlier (ie, aged 51 or older in 1998).

### Measures

Demographic characteristics (age, sex, race/ethnicity, education, and marital status) were obtained from 1998 HRS data. Age and education were measured as continuous variables. Race/ethnicity was categorized as non-Hispanic white, non-Hispanic black, and Hispanic/other. Marital status was measured as a dichotomous variable (married/partnered or other).

Measures of clinical conditions, including the number of self-reported physical or psychological chronic conditions and the duration of diabetes, were obtained from 2000 HRS data. The number of physical or psychological chronic diseases was the sum of indicators for whether a participant had ever been informed by a doctor that he or she ever had high blood pressure, diabetes, cancer, lung disease, heart disease, stroke, psychiatric problems, or arthritis. The duration of diabetes was calculated as the number of years between individuals' diagnosis of diabetes and the year 2000. Information about the age of diagnosis of diabetes was obtained from the question, "At what age were you told by a doctor that you had diabetes?"

Information about treatment modality was obtained by participants' answers to 3 questions from the 2000 HRS: "Are you following a special diet?," "In order to treat or control your diabetes, are you now taking medication that you swallow?," and "Are you now using insulin shots or a pump?" Participants who answered yes to the diet question but did not report using oral medications or insulin were placed in the "diet only" treatment group, participants who reported using oral medications only or a combination of oral medication and diet but did not report using insulin were placed in the "oral medication" treatment group, and participants who reported using insulin only or in combination with other regimens were placed in the "insulin only or combination" treatment group.

Self-reported information obtained in the 2000 HRS about lifestyle behaviors related to physical activity, substance use, and control of body weight were used. To report physical activity, participants were asked the yes/no question, "On average over the last 12 months have you participated in vigorous activity or exercise 3 times a week or more?" Substance use was assessed by asking, "Do you smoke cigarettes now?," "Have you ever felt that you should cut down on drinking?," "Have people ever annoyed you by criticizing your drinking?," "Have you ever felt bad or guilty about drinking?," or "Have you ever taken a drink first thing in the morning to steady your nerves or get rid of a hangover?" Participants who answered no to all questions were defined as "no substance use"; all others were defined as "substance use." Body mass index (BMI) was used to determine control of body weight. Participants were categorized as either having body weight control (BMI <30 kg/m^2^) or not (BMI ≥30 kg/m^2^), on the basis of National Heart, Lung, and Blood Institute guidelines (20). We recoded the responses to the above behaviors into 3 categories for lifestyle: poor (0 positive health behaviors), fair (1 positive health behavior), and good (≥2 positive health behaviors).

Glycemic control was determined by HbA1c levels. This biomarker was available through blood spot assays collected in the HRS 2003 Diabetes Study.

### Data analysis

SAS version 9.1 (SAS Institute, Inc, Cary, North Carolina) was used to analyze the data. To characterize the sample across major study variables and to test their association with HbA1c levels, we performed *t* tests, analysis of variance (ANOVA), and simple regression. Hierarchical multiple regression analyses were conducted to answer the study's specific research questions. Significance was set at *P* < .05. In model 1, only demographic variables (age and race/ethnicity) were entered as explanatory variables; in model 2, we added clinical condition variables (number of chronic diseases, duration of diabetes); in model 3, we added treatment modality variables (diet only, oral medication, or insulin use); and in model 4, we added a lifestyle variable (fair, good, or poor). Significance tests for *R* ^2^ increment were calculated to provide information on how well the sequential variables accounted for the variance in HbA1c levels beyond that accounted for by variables in the preceding model. To explore possible differences in the predictive patterns between middle-aged and older adults, the models were tested for the entire sample and separately for the middle-aged and older adult samples.

## Results

### Sample characteristics and bivariate analysis with HbA1c levels

Glycemic control was significantly associated with age, race/ethnicity, number of chronic diseases, duration of diabetes, treatment modality, and lifestyle (Table 1). Age was negatively correlated with HbA1c values. Participants who were non-Hispanic white, reported fewer chronic diseases, reported a shorter duration of diabetes, reported using diet-only treatment, or reported having good lifestyle behaviors had lower HbA1c levels. Sex, education, and marital status were not significantly associated with HbA1c levels and were excluded from the hierarchical regression analyses.

### Effects of demographics, clinical conditions, treatment modality, and lifestyle on glycemic control

Model 1, which included only age and race/ethnicity as variables, explained 4.8% of the variance in HbA1c levels (Table 2). Age was negatively associated with HbA1c levels, and non-Hispanic black and Hispanic participants had higher HbA1c levels than non-Hispanic white participants.

Model 2, which added duration of diabetes and the number of chronic diseases as variables, explained 7.6% of the variance in HbA1c levels. A significance test of *R* ^2^ increment from model 1 to model 2 suggests that the clinical conditions, over and above the demographic determinants, explained a significant proportion of the variance in HbA1c levels. Participants who reported having more chronic diseases or a longer duration of diabetes had higher HbA1c levels than participants who reported having fewer chronic diseases or a shorter duration of diabetes, independent of the demographic determinants.

Model 3, which added treatment modality as a variable, explained 14.1% of the variance in HbA1c levels. The change in *R* ^2^ from model 2 to model 3 was significant, suggesting that treatment modality has a substantial effect in predicting HbA1c levels, independent of demographic and clinical characteristics. Compared with participants who used insulin only or in combination with other regimens, participants who were treated with diet only or oral medications had lower HbA1c levels.

Although demographic characteristics, clinical conditions, and treatment modality explained an appreciable amount of the variance in HbA1c levels, an additional 2.1% of the variance was explained independently and significantly by lifestyle behaviors. An increase of 1 healthy behavior was associated with a decrease in HbA1c levels of more than 1 percentage point.

### Comparison of predictive patterns in middle-aged and older adults

In middle-aged adults, our selected predictors explained 21.3% of the total variance in HbA1c levels (Table 3). Demographics and clinical conditions accounted for the largest explained variance. Treatment modality also explained a significant proportion of the variance in HbA1c levels, as did lifestyle, which was added to model 4. The result suggests that, among middle-aged adults with type 2 diabetes, lifestyle behaviors are strongly associated with HbA1c levels, independent of the effects of demographic background, clinical conditions, and treatment modality.

In the older adult group, our selected predictors explained 10.2% of the variance in HbA1c levels, approximately half that in the middle-aged adult sample (Table 4). Demographics, clinical conditions, and lifestyle variables did not significantly predict HbA1c levels in older adults, but treatment modality did. The patterns of the 4 predictors on glycemic control in the entire sample, as well as in middle-aged adults and older adults separately, are presented in the Figure.

**Figure 1:**
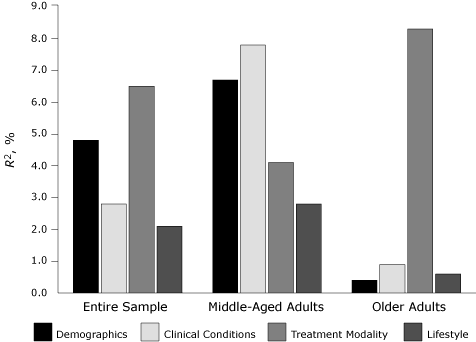
Effects of demographic factors, clinical conditions, treatment modality, and lifestyle on HbA1c levels among middle-aged adults (n = 379), older adults (n = 430), and the entire sample (N = 809), the 1998 and 2000 Health and Retirement Study (HRS) and the HRS 2003 Diabetes Study

**Table:** 

**Population**	***R* ^2^, %**			
	**Demographics**	**Clinical Conditions**	**Treatment Modality**	**Lifestyle**
Entire sample	4.8	2.8	6.5	2.1
Middle-aged adults	6.7	7.8	4.1	2.8
Older adults	0.4	0.9	8.3	0.6

## Discussion

Our results suggest that distinct predictive patterns of glycemic control exist for middle-aged and older adults and confirm the long-term beneficial link between general lifestyle behaviors and HbA1c levels, especially in middle-aged adults. The results also highlight the crucial role of medication treatment in older adults with type 2 diabetes.

The result that nonwhite adults with type 2 diabetes were less likely to achieve adequate glycemic control than were their white counterparts is in accord with other studies (4,10,11,21) and confirms the previous finding that mechanisms beyond our model covariates of clinical conditions, treatment modality, and general lifestyle underlie racial/ethnic disparities in glycemic outcomes. Our finding that younger age was associated with worse glycemic control is congruent with a previous study (17) and echoes other studies that suggest that early-onset type 2 diabetes is associated with worse glycemic control outcomes than is later-onset type 2 diabetes (22). Our finding that the number of chronic diseases and duration of diabetes, independent of demographics, predicted glycemic control is consistent with at least 1 study (12). Although our results showed that diet-only therapy was associated with lower HbA1c levels and that using insulin only or in combination with other regimens is associated with higher HbA1c levels, these relationships more likely represent a marker of diabetes severity than of medication effects themselves.

Of particular interest is the fact that demographic factors, clinical conditions and treatment modality explained a substantial proportion of the variance in HbA1c levels and that lifestyle behaviors independently explained 2.1% of the variance in HbA1c levels in the entire sample. The effect did not vary across sex and racial/ethnic groups, diabetes duration, number of chronic conditions, and diabetes treatment modality. The finding that an increase of 1 healthy behavior is associated with a decrease in HbA1c levels of more than 1 percentage point is not only significant but also clinically relevant.

Comparison of the predictive patterns between middle-aged adults and older adults reveals that in midlife, demographic factors and clinical conditions are the most substantial predictors for HbA1c levels. This finding has practical implications for diabetes care. Early interventions may be needed to help socioeconomically and clinically disadvantaged middle-aged adults with type 2 diabetes to achieve satisfactory glycemic control. Conversely, our finding that glycemic control in older age is especially sensitive to treatment modality suggests that diabetes care should also focus on older adults who exhibit poor adherence to medication recommendations. Furthermore, the lower explanatory power of our model among older adults compared with that among middle-aged adults implies that in older age, glycemic control may be affected by more complex factors than during midlife. This finding supports the increasing body of literature advocating special considerations for older adults with diabetes (23-26).

Our study has several limitations. First, although previous studies have found that the validity of self-reported exercise behavior is high among older adults (27), the validity of self-report measures of substance use and weight control among older adults remains uncertain. Rates of performing healthy behaviors may have been overestimated because of social desirability bias (28,29). Furthermore, past research with the HRS has shown that, on average, survey respondents are healthier than nonrespondents (30); therefore, the relationship between modifiable predictors and glycemic control may appear stronger than it actually is. Second, although our prospective design allowed us to investigate predictors of HbA1c levels, the lack of baseline HbA1c data limited our ability to infer causal effects of our predictors of glycemic control. Third, we may have underestimated the explanatory effects of general lifestyle behaviors because other general lifestyle factors, such as psychologically related behaviors (eg, stress management, self-control) were not included. Fourth, the comparison of the predictive effects of some explanatory variables on glycemic control in the 2 age groups may be confounded with a "survival effect." For example, less healthy members of minority groups may have died at earlier ages, leaving those surviving to age 65 or older as relatively healthier, thus potentially underestimating the effects of race/ethnicity on glycemic control in older age (30).

Overall, our results suggest that general lifestyle behaviors have a beneficial effect on glycemic control, beyond effects accounted for by demographic factors, clinical conditions, and treatment modality, especially in middle-aged adults. Our findings provide support for current diabetes care guidelines that recommend a lifestyle regimen across the entire span of diabetes care and highlight the need to help sociodemographically or clinically disadvantaged middle-aged adults with type 2 diabetes and older adults who exhibit poor adherence to medication recommendations to achieve glycemic control.

## Figures and Tables

**Table 1:** Sample Characteristics and Bivariate Association With HbA1c Levels, Middle-Aged (51-64 y) and Older Adults (≥65 y) With Type 2 Diabetes (N = 809), the 1998 and 2000 Health and Retirement Study (HRS) and the HRS 2003 Diabetes Study

Characteristics^a^	%^b^	Mean HbA1c Level,^c^ %	*P* Value^d^
**Mean age (range, 51-89 y)**	65.3	NA	<.001
**Sex**			
Male	50.0	7.27	.84
Female	50.0	7.29	
**Race/ethnicity**			
Non-Hispanic white	81.1	7.15	<.001
Non-Hispanic black	10.8	7.82	
Hispanic/other	8.2	7.72	
**Mean years of education (range, 0-17 y)**	11.8	NA	.91
**Marital status**			
Married/partnered	69.2	7.25	.41
Other	30.8	7.34	
**Mean no. of chronic diseases^e^**	3.0	NA	<.001
**Mean duration of diabetes (range, 1-42 y)**	9.9	NA	<.001
**Treatment modality**			
Diet only	28.1	6.75	<.001
Oral medication	50.9	7.33	
Insulin only or combination	21.0	7.86	
**Lifestyle^f^**			
Poor	3.2	8.54	<.001
Fair	34.4	7.33	
Good	62.3	7.18	

Abbreviations: HbA1c, hemoglobin A1c; NA, not applicable.

a Data for mean age, sex, race/ethnicity, mean years of education, and marital status are from the 1998 HRS. Data for all other characteristics are from the 2000 HRS.

b All values are percentages unless otherwise indicated.

c Data from the 2003 Health and Retirement Study Diabetes Survey.

d *P* values calculated using *t *test, analysis of variance (ANOVA), and simple regression; significance set at α = .05.

e The number of chronic diseases was the sum of indicators for whether a participant had ever been informed by a doctor that he or she ever had high blood pressure, diabetes, cancer, lung disease, heart disease, stroke, psychiatric problems, or arthritis.

f Poor = 0 positive health behaviors, fair = 1 positive health behavior, good ≥2 positive health behaviors.

**Table 2:** Hierarchical Regression Analyses Predicting HbA1c Levels in Middle-Aged (51-64 y) and Older Adults (≥65 y) With Type 2 Diabetes (N = 809), the 1998 and 2000 Health and Retirement Study (HRS) and the HRS 2003 Diabetes Study^a^

Variable	Model 1^b^		Model 2^c^		Model 3^d^		Model 4^e^	
	β	*P* Value	β	*P* Value	β	*P* Value	β	*P* Value
**Demographics**								
Age	−.02	<.001	−.03	<.001	−.02	<.001	−.02	<.001
Race/ethnicity								
Non-Hispanic black	.67	<.001	.57	<.001	.42	.008	.45	.005
Hispanic	.56	.002	.55	.002	.46	.001	.50	.004
Non-Hispanic white	1 [Reference]		1 [Reference]		1 [Reference]		1 [Reference]	
**Clinical Conditions**								
No. of chronic diseases	—	—	.09	.03	.04	.32	.04	.32
Duration of diabetes	—	—	.02	<.001	.01	.32	.01	.32
**Treatment Modality**								
Diet only	—	—	—	—	−1.17	<.001	−1.22	<.001
Oral medication	—	—	—	—	−.50	<.001	−.55	<.001
Insulin only or combination	—	—	—	—	1 [Reference]		1 [Reference]	
**Lifestyle^f^**								
Fair	—	—	—	—	—	—	−1.21	<.001
Good	—	—	—	—	—	—	−1.02	<.001
Poor	—	—	—	—	—	—	1 [Reference]	
**F^g^**	13.16	<.001	12.42	<.001	17.59	<.001	16.06	<.001
***R* ^2^, %**	4.80	—	7.62	—	14.09	—	16.18	—
**Δ*R* ^2^, %**	—	—	2.82	—	6.47	—	2.09	—
**F^h^**	—	—	34.89	<.001	30.12	<.001	9.95	<.001

Abbreviation: HbA1c, hemoglobin A1c.

a *P* values calculated using *t *test, analysis of variance (ANOVA), and simple regression; significance set at α = .05.

b Model 1: demographic variables only.

c Model 2: variables are demographics and clinical conditions.

d Model 3: variables are demographics, clinical conditions, and treatment modality.

e Model 4: variables are demographics, clinical conditions, treatment modality, and lifestyle.

f Poor = 0 positive health behaviors, fair = 1 positive health behavior, good ≥2 positive health behaviors.

g F test for model.

h F test for change in *R* ^2^.

**Table 3:** Hierarchical Regression Analyses Predicting HbA1c Levels in Middle-Aged Adults (51-64 y) With Type 2 Diabetes (N = 379), the 1998 and 2000 Health and Retirement Study (HRS) and the HRS 2003 Diabetes Study^a^

Variable	Model 1^b^		Model 2^c^		Model 3^d^		Model 4^e^	
	β	*P* Value	β	*P* Value	β	*P* Value	β	*P* Value
**Demographics (Race/Ethnicity)**								
Non-Hispanic black	1.13	<.001	.96	<.001	.82	.001	.80	.002
Hispanic	.88	<.001	.87	.004	.74	.01	.79	.007
Non-Hispanic white	1 [Reference]		1 [Reference]		1 [Reference]		1 [Reference]	
**Clinical Conditions**								
No. of chronic diseases	—	—	.24	<.001	.19	.002	.18	.003
Duration of diabetes	—	—	.04	<.001	.01	.32	.02	.046
**Treatment Modality**								
Diet only	—	—	—	—	−1.07	<.001	−1.18	<.001
Oral medication	—	—	—	—	−.35	.11	−.46	.04
Insulin only or combination	—	—	—	—	1 [Reference]		1 [Reference]	
**Lifestyle^f^**								
Fair	—	—	—	—	—	—	−1.22	<.001
Good	—	—	—	—	—	—	−.96	.008
Poor	—	—	—	—	—	—	1 [Reference]	
**F^g^**	12.90	<.001	14.38	<.001	12.82	<.001	11.40	<.001
***R* ^2^, %**	6.67	—	14.44	—	18.49	—	21.30	—
**Δ*R* ^2^, %**	—	—	7.77	—	4.05	—	2.81	—
**F^h^**	—	—	16.98	<.001	9.24	<.001	6.61	.001

Abbreviation: HbA1c, hemoglobin A1c.

a *P* values calculated using *t *test, analysis of variance (ANOVA), and simple regression; significance set at α = .05.

b Model 1: demographic variables only.

c Model 2: variables are demographics and clinical conditions.

d Model 3: variables are demographics, clinical conditions, and treatment modality.

e Model 4: variables are demographics, clinical conditions, treatment modality, and lifestyle.

f Poor = 0 positive health behaviors, fair = 1 positive health behavior, good ≥2 positive health behaviors.

g F test for model.

h F test for change in *R* ^2^.

**Table 4:** Hierarchical Regression Analyses Predicting HbA1c Levels in Older Adults (≥65 y) With Type 2 Diabetes (N = 430), the 1998 and 2000 Health and Retirement Study (HRS) and the HRS 2003 Diabetes Study^a^

Variable	Model 1^b^		Model 2^c^		Model 3^d^		Model 4^e^	
	β	*P* Value	β	*P* Value	β	*P* Value	β	*P* Value
**Demographics**								
**Race/ethnicity**								
Non-Hispanic black	0.13	.49	.17	.37	.09	.32	.11	.56
Hispanic	.24	.25	.27	.20	.24	.23	.25	.21
Non-Hispanic white	1 [Reference]		1 [Reference]		1 [Reference]		1 [Reference]	
**Clinical Conditions**								
No. of chronic diseases	—	—	−.03	.45	−.07	.08	−.06	.13
Duration of diabetes	—	—	.01	.32	−.01	.32	−.01	.32
**Treatment Modality**								
Diet only	—	—	—	—	−1.01	<.001	−1.07	<.001
Oral medication	—	—	—	—	−.46	.001	−.46	.001
Insulin only or combination	—	—	—	—	1 [Reference]		1 [Reference]	
**Lifestyle^f^**								
Fair	—	—	—	—	—	—	−.90	.13
Good	—	—	—	—	—	—	−.80	.18
Poor	—	—	—	—	—	—	1 [Reference]	
**F^g^**	.80	.45	1.38	.25	7.18	<.001	5.71	<.001
***R* ^2^, %**	.38	—	1.34	—	9.59	—	10.17	—
**Δ*R* ^2^, %**	—	—	.96	—	8.25	—	0.58	—
**F^h^**	—	—	2.07	.13	19.30	<.001	1.36	.26

Abbreviation: HbA1c, hemoglobin A1c.

a *P* values calculated using *t *test, analysis of variance (ANOVA), and simple regression; significance set at α = .05.

b Model 1: demographic variables only.

c Model 2: variables are demographics and clinical conditions.

d Model 3: variables are demographics, clinical conditions, and treatment modality.

e Model 4: variables are demographics, clinical conditions, treatment modality, and lifestyle.

f Poor = 0 positive health behaviors, fair = 1 positive health behavior, good ≥2 positive health behaviors.

g F test for model.

h F test for change in *R* ^2^.
